# Electron microscopy ascertains presence of centrioles in rectal epithelial cells of *C. elegans* L1 larvae

**DOI:** 10.17912/micropub.biology.001624

**Published:** 2025-05-22

**Authors:** Marie Croisier, Coralie Busso, Nils Kalbfuss, Alexander Woglar, Graham Knott, Pierre Gönczy

**Affiliations:** 1 Bio-EM platform, School of Life Sciences, Swiss Federal Institute of Technology Lausanne (EPFL), Lausanne, Switzerland; 2 Swiss Institute for Experimental Cancer Research (ISREC), School of Life Sciences, Swiss Federal Institute of Technology Lausanne (EPFL), Lausanne, Switzerland

## Abstract

Centrioles are microtubule-based organelles important for cellular organization and function. *C. elegans* embryos undergo extensive programmed centriole elimination, with merely 7 post-mitotic cells retaining a focus of the centriolar proteins SAS-4 and SAS-7 in L1 larvae. Here, we addressed whether such foci correspond to *bona fide* centrioles by conducting serial-section electron microscopy. Our analysis ascertains that centriolar microtubule configurations are indeed present in the B, F, U and Y rectal epithelial cells. Therefore, centrioles are truly spared from elimination in these cells, calling for investigating the importance of such retention compared to the elimination program occurring in most post-mitotic cells.

**Figure 1. Electron microscopy identifies centrioles in rectal epithelial cells of L1 larvae:**
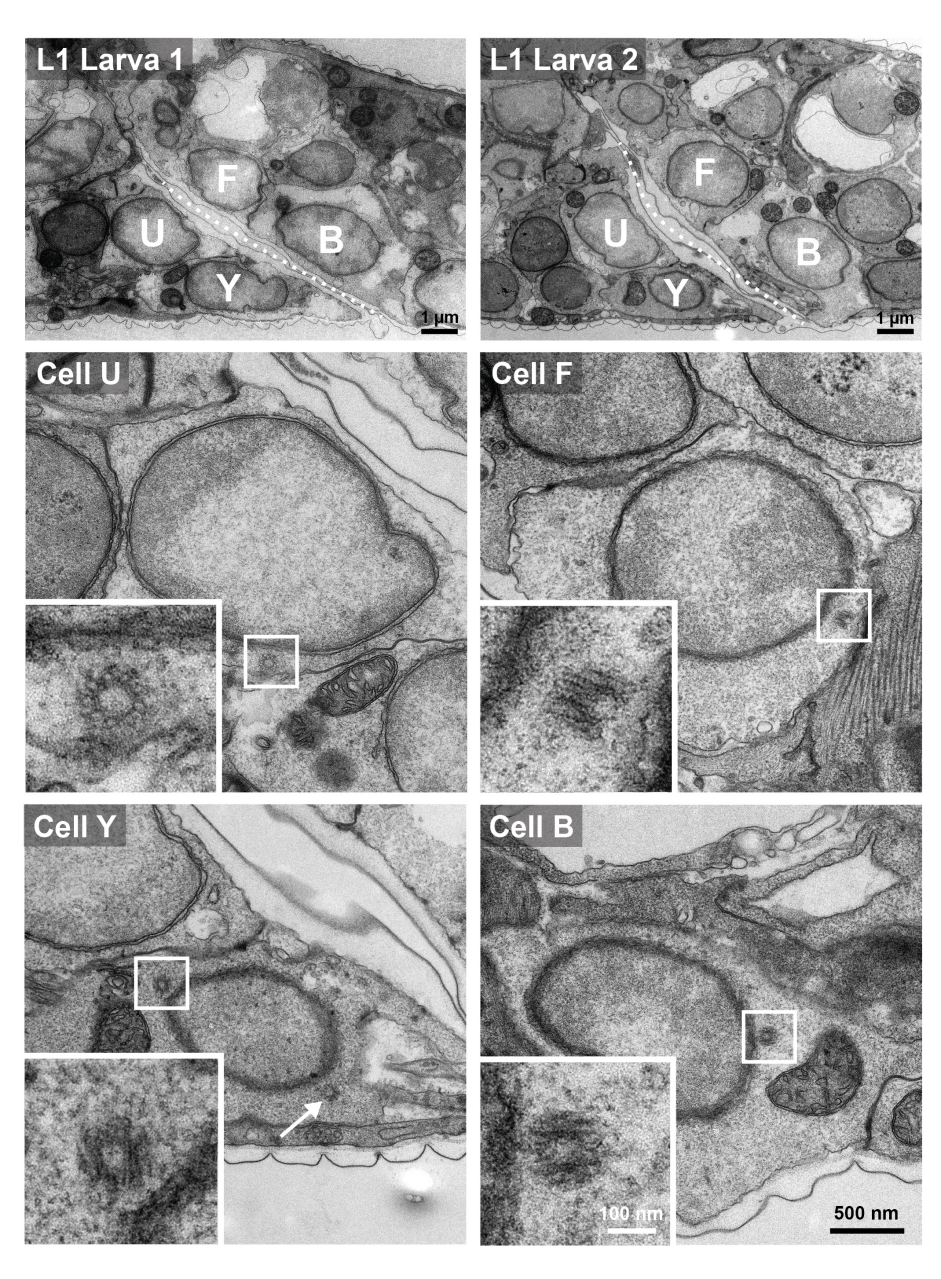
(top row) Overview of rectal region in the two L1 larvae (left: Larva 1, right: Larva 2) that were serially-sectioned for EM, with an indication of the four rectal epithelial cells analyzed. Note rectal slit (dotted line), with U and Y on the left (anterior), F and B on the right (posterior). (middle and bottom rows): 50 nm EM section of indicated cells (U from Larva 1; F, Y and B from Larva 2). Square indicates position of magnified inset shown on bottom left of each panel; arrow in Y points to second centriole visible in the same section in this case.

## Description

Centrioles are minute microtubule-based organelles important for cell signaling, motility and division across eukaryotes (reviewed in Winey and O'Toole, 2014). In general, there are two centrioles early in the cell cycle; each centriole then mentors the assembly of another centriole in its vicinity, so that four centrioles are present during mitosis, with two being destined to each resulting daughter cell (reviewed in Firat-Karalar and Stearns, 2014). Pioneering work conducted in *C. elegans* led to the identification of a small set of proteins essential for centriole assembly: SAS-7 , SPD-2 , ZYG-1 , SAS-6 , SAS-5 and SAS-4 (Dammermann et al., 2004; Delattre et al., 2004; Kemp et al., 2004; Kirkham et al., 2003; Leidel and Gönczy, 2003; Leidel et al., 2005; O'Connell et al., 2001; Pelletier et al., 2004; Sugioka et al., 2017). Homologues of most of these proteins are likewise critical for centriole assembly in other eukaryotic species. Although centrioles are generally very stable, they can be eliminated in some physiological circumstances, including during oogenesis (reviewed in Kalbfuss and Gönczy, 2023b; Werner et al., 2017). Centrioles can also be eliminated during early development: using the presence of SAS-4::GFP and GFP::SAS-7 foci as proxies for centrioles, it was established that ~88% of cells undergo extensive programmed centriole elimination during *C. elegans* embryogenesis (Kalbfuss and Gönczy, 2023a). Live imaging and lineage analysis revealed that centriole elimination is stereotyped, occurring at a given time in a given cell type. Besides cells that later proliferate or endoreduplicate, only 7 post-mitotic cells retain centrioles throughout embryogenesis as judged by the presence of SAS-4 ::GFP and GFP:: SAS-7 foci in L1 larvae: the rectal epithelial cells B, F, U, Y and K', as well as the amphid socket cell AMsoL and the sheath cell AMsoR (Kalbfuss and Gönczy, 2023a). In addition, these cells retain RFP:: SPD-2 , as well as GFP:: PCMD-1 and GFP:: SPD-5 , which mark the pericentriolar material (PCM) surrounding centrioles (Kalbfuss et al., 2023). However, whether these post-mitotic cells harbor *bona fide* centrioles or merely centriolar and PCM proteins that remain localized in a focus is not known. That the latter may be the case is suggested by the fact that, during oogenesis centriole elimination in *C. elegans* , foci of centriolar proteins remain present much longer than the organelle itself, as monitored by electron microscopy (EM) or Ultrastructure Expansion coupled to STED super-resolution microscopy (U-Ex-STED) (Pierron et al., 2023). Moreover, most rectal epithelial cells no longer have a focus of SAS-4 ::GFP in subsequent larval stages or in the adult (Kalbfuss et al., 2023), raising the possibility that *bona fide* centrioles may not be present in these cells even in L1 larvae.

Given the above considerations, we set out to conduct serial section EM analysis to unequivocally address whether *bona fide* centrioles are present in the four clearly recognizable rectal epithelial cells positioned on either side of the rectal slit in L1 larvae: B, F, U, and Y ( Figure 1, top row) (Sulston and Horvitz, 1977). Centrioles in *C. elegans* are merely ~120 nm x ~150 nm in dimensions (Sugioka et al., 2017; Woglar et al., 2022), and are usually located in the vicinity of the nucleus, thereby enabling their detection in EM experiments despite their small size. Here, L1 larvae were collected, cut into segments to facilitate fixation, and then processed for serial-section EM (Methods). We collected 50 nm serial sections in two larval tail segments across the nucleus of B, F, U, and Y ( Figure 1, top row). Given that these cells are post-mitotic, two centrioles are expected to be present, and we found this to be the case indeed in all 8 cells examined (exemplified in Figure 1, middle and bottom rows, where insets show high magnification views of one centriole in each cell type). We conclude that *bona fide* centrioles are truly spared from elimination in these four rectal epithelial cells in L1 larvae. Intriguingly, cells that correspond to B, F, U and Y are proliferative in the male (Sulston and Horvitz, 1977), a relationship that might explain why centrioles remain present in the rectal epithelial cells of the hermaphrodite. Regardless of the reason, it will be interesting to investigate the mechanisms through which centrioles are retained in B, F, U and Y at this stage, whereas the vast majority of post-mitotic cells have become devoid of the organelle by then, and why such retention may be of functional importance.

## Methods

Glass-bottom Petri dishes were coated with a solution of poly-D-lysine (2 mg/ml in H₂O, Sigma P1024-10MG), left to dry for 10–30 min, and then washed three times with distilled water, followed by ethanol before being dried completely. Synchronized L1 larvae of genotype *sas-7* ( *or1940* [ *gfp:: sas-7* ])III; *glo-1* ( *zu931* )X, *gaIs245* [ *col-34p:: HIS-24 ::mCherry; unc-119 (+)* ] V (Kalbfuss and Gönczy, 2023a) were collected in M9 buffer, the excess removed, and then mixed with a fixative solution (0.9% paraformaldehyde and 0.1% glutaraldehyde in PBS buffer -0.01M, pH 7.0). Larvae were then cut into segments using a scalpel blade (No. 10, Swann-Morton) and left in the fixative for 5 min. The mixture of larval segments and fixative was transferred into the glass-bottom Petri dishes and spun down at 1000 rpm to promote adhesion to the glass.

The procedure of staining and embedding for EM was the same as described (Kalbfuss and Gönczy, 2023a), and is as follows. The solution was exchanged for another fixation solution of 1 % glutaraldehyde and 0.9 % paraformaldehyde in 0.05 M cacodylate buffer (pH 7.4); the Petri dishes were then sealed with parafilm and stored overnight at 4°C for staining and embedding the following day.

The following procedure was performed in the Petri dishes. The larval segments were washed three times in cacodylate buffer (0.05 M cacodylate buffer with 0.09 M sucrose, pH 7.0; 5 min for each wash), and then stained for 40 min in 1.0 % osmium tetroxide with 0.8 % potassium ferrocyanide in cacodylate buffer 0.1M pH 7.0, followed by 15 min in 0.2 % tannic acid (in 0.5 M cacodylate buffer, pH 7.0). They were then washed in the same buffer, followed by distilled water, and then sodium acetate at pH 5.2 for 5 min. Larval segments were given a final stain of 1 % uranyl acetate in sodium acetate (pH 5.2), and washed in the buffer alone (3 times for 5 min each). Then, larval segments were washed in distilled water, dehydrated with increasing concentrations of ethanol and once in 100 % ethanol; increasing concentrations of Epon resin (Embed 812 embedding kit, EMS) were introduced until 100%. The Petri dishes were then filled with resin to a depth of resin of 2 mm, before being transferred to an oven at 65°C, and left there for 24 h to allow the resin to harden.

Following hardening, larval tail segments of interest were cut from the flat sheet of resin and glued onto a blank resin block using acrylic resin. This was then trimmed using glass knives, mounted in an ultramicrotome (Leica Microsystems UC6). Once a small trapezoid block (400 x 100 µm approximate dimensions) had been made, serial sections were cut at 50 nm thickness with a diamond knife (Diatome), and collected onto single slot copper grids with a pioloform support film. These were contrasted with 2% lead citrate and 1% uranyl acetate, and images taken with a transmission EM at 80 kV (Tecnai Spirit, FEI Company with an Eagle CCD camera).

## References

[R1] Dammermann A, Müller-Reichert T, Pelletier L, Habermann B, Desai A, Oegema K (2004). Centriole assembly requires both centriolar and pericentriolar material proteins.. Dev Cell.

[R2] Delattre M, Leidel S, Wani K, Baumer K, Bamat J, Schnabel H, Feichtinger R, Schnabel R, Gönczy P (2004). Centriolar SAS-5 is required for centrosome duplication in C. elegans.. Nat Cell Biol.

[R3] Fırat-Karalar EN, Stearns T (2014). The centriole duplication cycle.. Philos Trans R Soc Lond B Biol Sci.

[R4] Kalbfuss N, Gönczy P (2023). Extensive programmed centriole elimination unveiled in C. elegans embryos.. Sci Adv.

[R5] Kalbfuss N, Gönczy P (2023). Towards understanding centriole elimination.. Open Biol.

[R6] Kalbfuss N, Berger A, Gönczy P (2023). Mapping of centriolar proteins onto the post-embryonic lineage of C.&nbsp;elegans.. Dev Biol.

[R7] Kemp CA, Kopish KR, Zipperlen P, Ahringer J, O'Connell KF (2004). Centrosome maturation and duplication in C. elegans require the coiled-coil protein SPD-2.. Dev Cell.

[R8] Kirkham M, Müller-Reichert T, Oegema K, Grill S, Hyman AA (2003). SAS-4 is a C. elegans centriolar protein that controls centrosome size.. Cell.

[R9] Leidel S, Gönczy P (2003). SAS-4 is essential for centrosome duplication in C elegans and is recruited to daughter centrioles once per cell cycle.. Dev Cell.

[R10] O'Connell KF, Caron C, Kopish KR, Hurd DD, Kemphues KJ, Li Y, White JG (2001). The C. elegans zyg-1 gene encodes a regulator of centrosome duplication with distinct maternal and paternal roles in the embryo.. Cell.

[R11] Pelletier L, Ozlü N, Hannak E, Cowan C, Habermann B, Ruer M, Müller-Reichert T, Hyman AA (2004). The Caenorhabditis elegans centrosomal protein SPD-2 is required for both pericentriolar material recruitment and centriole duplication.. Curr Biol.

[R12] Pierron M, Woglar A, Busso C, Jha K, Mikeladze-Dvali T, Croisier M, Gönczy P (2023). Centriole elimination during Caenorhabditis elegans oogenesis initiates with loss of the central tube protein SAS-1.. EMBO J.

[R13] Sugioka K, Hamill DR, Lowry JB, McNeely ME, Enrick M, Richter AC, Kiebler LE, Priess JR, Bowerman B (2017). Centriolar SAS-7 acts upstream of SPD-2 to regulate centriole assembly and pericentriolar material formation.. Elife.

[R14] Sulston JE, Horvitz HR (1977). Post-embryonic cell lineages of the nematode, Caenorhabditis elegans.. Dev Biol.

[R15] Werner S, Pimenta-Marques A, Bettencourt-Dias M (2017). Maintaining centrosomes and cilia.. J Cell Sci.

[R16] Winey M, O'Toole E (2014). Centriole structure.. Philos Trans R Soc Lond B Biol Sci.

[R17] Woglar A, Pierron M, Schneider FZ, Jha K, Busso C, Gönczy P (2022). Molecular architecture of the C. elegans centriole.. PLoS Biol.

